# Differential Effect of Visual and Proprioceptive Stimulation on Corticospinal Output for Reciprocal Muscles

**DOI:** 10.3389/fnint.2019.00063

**Published:** 2019-10-29

**Authors:** Takako Suzuki, Makoto Suzuki, Naohiko Kanemura, Toyohiro Hamaguchi

**Affiliations:** ^1^School of Health Sciences, Saitama Prefectural University, Saitama, Japan; ^2^Department of Rehabilitation, Faculty of Health Sciences, Tokyo Kasei University, Saitama, Japan

**Keywords:** corticospinal excitability, magnetic stimulation, reciprocal muscles, observation, vibration

## Abstract

This study investigated the corticospinal excitability of reciprocal muscles during tasks involving sensory difference between proprioceptive and visual inputs. Participants were instructed to relax their muscles and to observe a screen during vibratory stimulation. A video screen was placed on the board covering the right hand and forearm. Participants were randomly tested in four conditions: resting, control, static, and dynamic. The resting condition involved showing a black screen, the control condition, a mosaic patterned static videoclip; the static condition, a static videoclip of wrist flexion 0°; and the dynamic condition, a videoclip that corresponded to each participant’s closely-matched illusory wrist flexion angle and speed by vibration. Vibratory stimulation (frequency 80 Hz and duration 4 s) was applied to the distal tendon of the dominant right extensor carpi radialis (ECR) using a tendon vibrator in the control, static, and dynamic conditions. Four seconds after the vibratory stimulation (end of vibration), the primary motor cortex at the midpoint between the centers of gravity of the flexor carpi radialis (FCR) and ECR muscles was stimulated by transcranial magnetic stimulation (TMS). The ECR motor evoked potential (MEP) amplitudes significantly increased in the control condition compared to the resting condition, whereas the FCR MEP amplitudes did not change between the resting and control conditions. In addition, the ECR MEP amplitudes significantly increased in the static condition compared to the dynamic condition. However, the FCR MEP amplitudes significantly increased in the dynamic condition compared to the static condition. These results imply that the difference between visuo-proprioceptive information had an effect on corticospinal excitability for the muscle. In conclusion, we found that proprioceptive and visual information differentially altered the corticospinal excitability of reciprocal muscles.

## Introduction

Humans can perceive their limb position in space based on both proprioceptive and visual information (Hagura et al., 2007). Therefore, for accurate motor control, proprioceptive and visual information of the body and environment has to be integrated and transformed into an appropriate motor command (Reichenbach et al., 2014; Davare et al., 2015). However, there are inherent differences in the neural transmission and integration time for proprioceptive and visual information. Therefore, it is important to consider the impact of the difference between proprioceptive and visual information on human motor output based on the perception of limb position.

Previous studies (Casini et al., 2006; Kito et al., 2006; Hagura et al., 2007; Lapole and Tindel, 2015) have investigated the cortical areas activated during kinesthetic sensations based on proprioceptive information from Ia afferent neurons *via* muscle spindle input. These studies (Casini et al., 2006; Kito et al., 2006; Hagura et al., 2007; Lapole and Tindel, 2015) used the well-established tendon vibration paradigm in which mechanical stimulation elicits the perception of illusory joint movements through Ia afferent neurons *via* activation of muscle spindle receptors. The proprioceptive inputs by tendon vibration lead to both facilitation of spinal Ia-α loop excitability (Gandevia, 2001) and to cortical activities related to the kinesthetic illusion of joint movement (Casini et al., 2006; Hagura et al., 2007; Lapole and Tindel, 2015). Previous studies have found that the cortical areas associated with the kinesthetic illusion were the supplementary motor area (SMA), premotor cortex (PM), posterior parietal cortex, and primary motor cortex (M1; Münte et al., 1996; Naito et al., 1999; Naito and Ehrsson, 2001; Kaelin-Lang et al., 2002; Casini et al., 2006; Lapole and Tindel, 2015). However, proprioceptive information, including that of muscle spindle receptors, does not directly determine the absolute limb position (Burgess et al., 1982). Thus, when the limb position is estimated, the visual information is also available (Smeets et al., 2006; Hagura et al., 2007). Visual information during observation of movement activates the SMA, PM, superior parietal lobe, and M1, and these cortical areas are similar to the areas related to the actual physical execution of similar movements (Filimon et al., 2007; Wright et al., 2016). Additionally, previous studies (Ehrsson et al., 2004; Makin et al., 2008; Brozzoli et al., 2012; Gentile et al., 2013; Limanowski and Blankenburg, 2016) have noted that the PM and posterior parietal cortex perform multisensory integration between proprioceptive and visual information. These previous studies have suggested that the illusory sensation of joint movement based on proprioceptive input and the observation of joint movement based on visual input act by utilizing partially overlapping processing routes including the SMA, PM, and parietal cortex, and these brain areas influence M1 activity during visuo-proprioceptive tasks.

Moreover, previous studies have shown that the Ia inhibitory interneurons were activated by Ia afferent input from the agonist muscle (Tanaka, 1974; Day et al., 1984; Kagamihara and Tanaka, 1985). These studies have suggested that central volleys from the motor cortex to the motor neurons of the agonist muscle could facilitate Ia inhibitory interneurons or directly inhibit the antagonist muscle (Hoshiyama et al., 1996; Yang et al., 2006; Gerachshenko and Stinear, 2007; Giacobbe et al., 2011). It is likely that multisensory integration from any given cortical site related to proprioceptive and visual input will differently alter M1 activity, diverging into reciprocal muscles with different “gains” (Huntley and Jones, 1991; Melgari et al., 2008). Previous studies have found an increase in corticospinal excitability for the muscle stimulated by tendon vibration (Mancheva et al., 2014) and corticospinal excitability for observational agonist muscles related to observation of joint movement (Wright et al., 2016). However, other studies did not find changes in corticospinal excitabilities for either the illusory muscle (Lapole and Tindel, 2015) or the reciprocal muscle stimulated by tendon vibration (Mancheva et al., 2014). Therefore, although corticospinal excitabilities for agonist muscles increase, and those for antagonist muscles decrease in accordance with voluntary movements (Hoshiyama et al., 1996), the changes in corticospinal excitabilities for reciprocal muscles by visuo-proprioceptive information remain controversial. In fact, because previous studies (Casini et al., 2006; Kito et al., 2006; Filimon et al., 2007; Forner-Cordero et al., 2008; Mancheva et al., 2014; Lapole and Tindel, 2015; Wright et al., 2016) used either vibratory or observational information, it is impossible to know whether the corticospinal excitabilities of reciprocal muscles were differently altered by multisensory difference between proprioceptive and visual information. Therefore, although multisensory integration between the proprioceptive and visual information associated with the perception of limb position contains inherent differences and conflicts, researchers do not yet understand whether they affect corticospinal excitability of reciprocal muscles. Such data could help to elucidate the relationship between corticospinal excitability of reciprocal muscles and multisensory difference during visuo-proprioceptive stimulation. In addition to expanding on previous findings, exploring how proprioceptive and visual stimulations affect corticospinal excitabilities for reciprocal muscles may contribute to elucidating the integration of multisensory information for accurate motor control.

Because the temporal resolution of transcranial magnetic stimulation (TMS) is suitable for the observation of corticospinal excitability changes induced by proprioceptive and visual stimulations, we designed a paradigm involving artificial multisensory difference between kinesthetic sensation based on vibratory proprioceptive input and static or dynamic movement observation based on visual input for corticospinal excitabilities. This paradigm facilitates the investigation of corticospinal excitabilities of reciprocal muscles in the context of conflicting multisensory information. We predicted that if vibratory proprioceptive stimulation affects corticospinal reciprocal function, then corticospinal excitability for the vibratory stimulated muscle should increase, whereas that for the reciprocal non-stimulated muscle would decrease; additionally, if multisensory difference between illusory movement based on vibratory proprioceptive input and observation of movement based on visual input also affect corticospinal excitability, then corticospinal excitabilities for the muscle stimulated by tendon vibration and for the agonist muscle by observation of movement (reciprocal muscle by tendon vibration) should be differently changed by visuo-proprioceptive information difference. We, therefore, used TMS to investigate corticospinal excitation of reciprocal muscles during visuo-proprioceptive tasks with competing multisensory information.

## Materials and Methods

### Participants

We recruited 20 healthy participants [17 women and three men, aged 20–21 years, mean ± standard deviation (SD): 21.2 ± 0.7 years] for the sensory recognition and corticospinal excitability measurements. All participants took part in the illusion-confirmation and in the multisensory-conflict experiments described below. No participant had risks of adverse events from TMS (Rossi et al., 2009) or use of medications or any psychiatric or neurological diseases. The mean laterality quotient score of the Edinburgh Handedness Inventory (Oldfield, 1971) was 0.9 points (SD: 0.2 points), and the right-handedness of the participants was confirmed. The Ethics Committee of the Saitama Prefectural University approved the experimental procedures, and the experiments were performed in accordance with the principles of the Declaration of Helsinki. Written informed consent was obtained from all participants.

### Illusion-Confirmation Experiment

#### Tendon Vibration

Previous studies (Collins and Prochazka, 1996; Kito et al., 2006) have noted that there are interindividual differences in the illusory perception of movement range and speed during vibratory stimulation. We, therefore, conducted an illusion-confirmation experiment to assess each participant’s perception of illusory movement range and speed by vibration. The participants were seated comfortably; the right arm was vertically beside the body, and the forearm remained in a neutral posture on a cushioned supporter. The forearm and wrist were relaxed throughout the experiment. During the illusion-confirmation trials, vibratory stimulation (frequency 80 Hz and duration 10 s) was applied to the distal tendon of the dominant right extensor carpi radialis muscle (ECR) using a tendon vibrator (MD011-YA, Daito Electric Machine Industry Co., Ltd., Osaka, Japan). The acme of the sphericity vibrator head was perpendicularly positioned at the center of the tendon of the ECR. During the illusion-confirmation trials, the participants were instructed to fully relax their muscles and to keep their eyes closed.

#### Assessment of Illusory Movement

**Figure 1:**
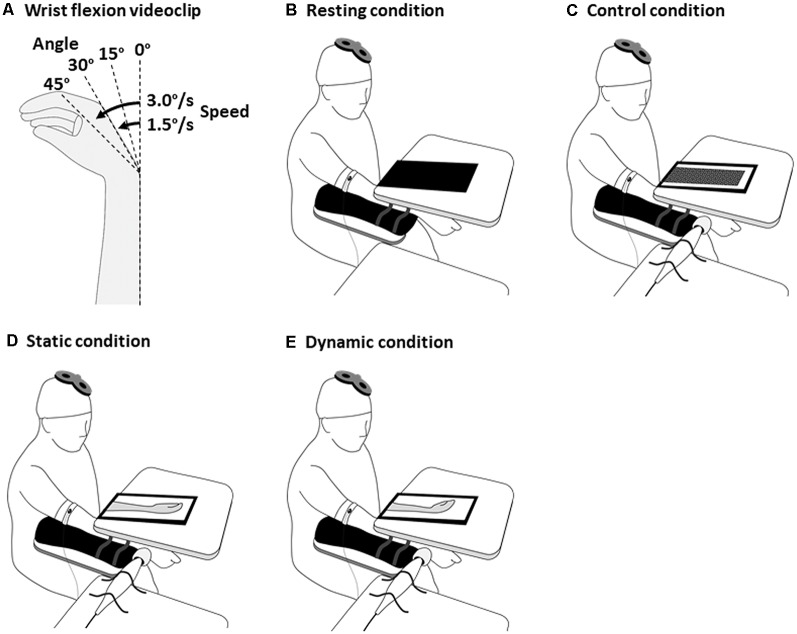
Experimental design for the tasks with visuo-proprioceptive information difference. The wrist flexion videoclips **(A)**, the resting condition **(B)** involved showing a black screen, the control condition **(C)**, a mosaic patterned static videoclip; the static condition **(D)**, a static videoclip of wrist flexion 0°; and the dynamic condition **(E)**, a videoclip that corresponded to each participant’s closely-matched wrist flexion angle and speed. The participants were instructed to fully relax their muscles. In the control, static, and dynamic conditions, the distal tendon of the dominant right ECR was stimulated by a tendon vibrator. Transcranial magnetic stimulation (TMS) was delivered to the primary motor cortex at the end of the vibratory stimulation. ECR, extensor carpi radialis.

A video screen was placed on the board covering the right hand and forearm. After the 10-s vibratory stimulation, the black screen was switched to videoclips. The videoclips randomly showed four wrist flexion angles (0°, 15°, 30°, and 40°), followed by two random movement speeds (1.5°/s and 3.0°/s; Figure 1A). Each videoclip required responses on a 5-point Likert scale, ranging from 1 to 5, with 1 indicating marked difference between the illusory and videoclip movements, and 5 indicating that the illusory and videoclip movements were closely matched.

### Multisensory-Conflict Experiment

#### Electromyographic (EMG) Recordings

Surface electromyography (EMG) activity was recorded from the flexor carpi radialis (FCR) and ECR muscles using double differential surface electrodes (FAD-DEMG1, 4Assist, Tokyo, Japan). The EMG signals were amplified ×100, bandpass filtered at 10–1,000 Hz with a DL-140 amplifier (4Assist, Tokyo, Japan), and digitized at 10 kHz with a PowerLab system (ADInstruments, Dunedin, New Zealand).

#### TMS

A figure-of-eight coil with a Magstim 200^2^ stimulator (Magstim, Whitland, UK) was used for TMS. A current was induced from the posterolateral to the anteromedial left brain. The coil handle was pointed dorsolaterally at approximately 45° to the midline, and the coil was maintained tangential to the scalp throughout the stimulation.

#### Motor Representational Map

On each participant’s head, a 6 × 6 cm^2^ grid of 25 positions (1.5-cm spacings) was marked, and its center was on the coordinates of (5.0 mm, 55.0 mm) from Cz in the international 10–20 system in reference to the midpoint between the center of gravities (CoGs) of the FCR and ECR (Suzuki et al., 2012, 2017). The resting motor threshold (RMT) was defined as the stimulus intensity elicited at least 50 μV MEPs for the resting FCR or ECR muscles in five out of 10 consecutive trials at the center of the 25 positions. Five MEPs evoked by 120% RMT were recorded for each scalp position (interstimulus interval: 5 s) in a clockwise spiral beginning at the center of the 25 positions. The CoG was calculated separately for each muscle with anteroposterior (*x*) and mediolateral (*y*) coordinates in reference to Cz as per the following formula (Marconi et al., 2011; Meesen et al., 2011; Suzuki et al., 2012, 2014a,b, 2017):

$$CoG=\left[\frac{\sum a_{i}x_{i}}{\sum a_{i}},\frac{\sum a_{i}y_{i}}{\sum a_{i}}\right],$$

where *x*_i_ and *y*_i_ are the stimulus coordinates, and *a*_i_ is the motor evoked potential (MEP) amplitude. The CoGs correspond to the locations of the most excitable neuron populations projecting to the target muscle. During the multisensory-conflict trials, we delivered a single-pulse TMS of 120% of the FCR’s RMT at the midpoint between the CoGs of the FCR and ECR.

#### Vibration and Observation

In the multisensory-conflict experiments, the participants were tested in four, randomly-ordered conditions: resting, control, static, and dynamic conditions (Figure 1). A 6-min inter-condition interval was set to diminish the kinesthetic aftereffect by the vibration (Kito et al., 2006). Vibratory stimulation (frequency 80 Hz and duration 4 s) was applied to the distal tendon of the dominant right ECR using a tendon vibrator (MD011-YA) in the control, static, and dynamic conditions. A video screen was placed on the board covering the right hand and forearm. The resting condition involved showing a black screen (Figure 1B), the control condition, a mosaic patterned static videoclip (Figure 1C); the static (conflict) condition, a static videoclip of wrist flexion 0° (Figure 1D); and the dynamic (non-conflict) condition, a videoclip that corresponded to each participant’s closely-matched illusory wrist flexion angle and speed in the illusion-confirmation experiment (Figure 1E). Previous experiments on changes in corticospinal excitability by vibratory stimulation (Kito et al., 2006; Forner-Cordero et al., 2008; Mancheva et al., 2014) used stimulation times ranging between 4 s and 25 s. In our illusion-confirmation experiment, the lowest wrist flexion angle and the highest wrist flexion speed were 15° and 3.0°/s, respectively. This indicated that the shortest wrist movement time was 5 s. Therefore, TMS was delivered 4 s after the vibratory stimulation because this allowed us to investigate corticospinal excitability without conflict between proprioceptive wrist flexion based on vibration, and visual wrist flexion based on observation during the illusory movement. Each condition was repeated for 20 trials at random 5–7-s intervals. The participants were instructed to fully relax their muscles and to observe the screen during the vibratory stimulation. Four seconds after the vibratory stimulation (end of vibration), a TMS of the FCR’s 120% RMT was delivered at the midpoint between the CoGs of the FCR and ECR.

### Data Analysis

In the illusion-confirmation experiment, we compared the Likert scale scores reflecting the illusory sensations of wrist flexion angles across 0°, 15°, 30°, and 40° using Friedman’s test and *post hoc* analysis with the Steel–Dwass test. Differences in illusory sensations of wrist flexion speeds of 1.5°/s and 3.0°/s were analyzed with the Wilcoxon signed-rank test. In the multisensory-conflict experiment, to clarify whether vibratory proprioceptive stimulation affects corticospinal reciprocal function, the MEP amplitudes of the ECR and FCR between the resting and control conditions were compared with the paired *t*-test. Subsequently, to clarify whether multisensory difference between illusory movement based on vibratory proprioceptive input and observation of movement based on visual input also affected corticospinal excitability, the MEP amplitudes in the static and dynamic conditions were normalized against those in the control condition. Normalized MEP amplitudes were calculated by dividing each MEP amplitude for ECR or FCR in the static and dynamic conditions by the mean MEP amplitude in the control condition in each participant’s ECR or FCR. The normalized MEP amplitudes of the static and dynamic conditions were then compared by paired *t*-test. Statistical significance was defined as *p* < 0.05. All statistical analyses were performed with R 3.5.2 (R Foundation for Statistical Computing, Vienna, Austria).

## Results

### Illusion-Confirmation Experiment

**Figure 2:**
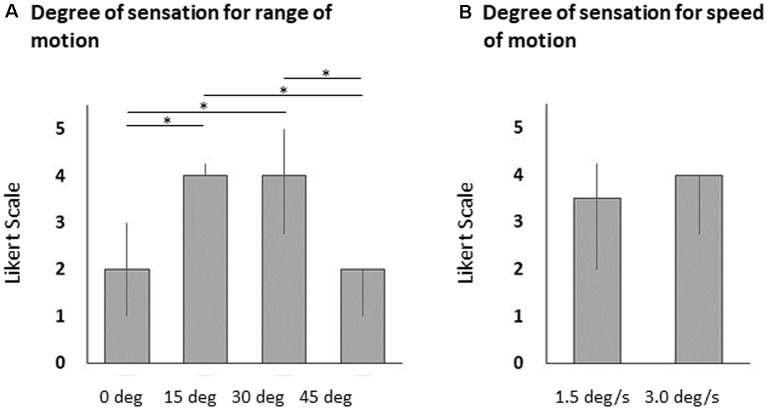
Bar graphs of the illusory sensations for wrist flexion angle **(A)** and flexion speed **(B)**. The columns and error bars denote the median and interquartile range, respectively. The illusory sensations of wrist flexion angles at 15° and 30° were significantly higher than those at 0° and 45° (Friedman’s test, *p < 0.0001 and Steel–Dwass test, 0° vs. 15°, *p < 0.0001, 0° vs. 30°, *p < 0.0001, 15° vs. 45°, *p < 0.0001, 30° vs. 45°, *p < 0.0001). However, the illusory sensations of wrist flexion speeds were small and were not significantly different between 1.5°/s, and 3.0°/s (Wilcoxon signed-rank test, *p* = 0.620).

Vibratory stimulation evoked illusory movements in all participants. The Likert scale scores associated with illusory sensations of wrist flexion angles at 15° and 30° were significantly higher than those at 0° and 45° (Friedman’s test, *p* < 0.0001 and Steel–Dwass test, 0° vs. 15°, *p* < 0.0001, 0° vs. 30°, *p* < 0.0001, 15° vs. 45°, *p* < 0.0001, 30° vs. 45°, *p* < 0.0001; Figure 2A). The difference in the Likert scale scores associated with illusory sensations of wrist flexion speeds were small and were not significantly different between 1.5°/s, and 3.0°/s (Wilcoxon signed-rank test, *p* = 0.620; Figure 2B). For 11 of 20 participants, the closely-matched illusory wrist flexion angle was 15° by the 80-Hz-vibratory stimulation, and for nine participants, the closely-matched illusory wrist flexion angle was 30° by the 80-Hz vibratory stimulation. For seven of 20 participants, the closely-matched illusory wrist flexion speed was 1.5°/s by the 80-Hz vibratory stimulation, and for 13 participants, the closely-matched illusory wrist flexion speed was 3.0°/s by the 80-Hz vibratory stimulation.

### Multisensory-Conflict Experiment

The CoGs for the FCR and ECR muscles were located at (5.2 ± 7.4 mm, 52.8 ± 4.9 mm) and (5.8 ± 8.2 mm, 52.1 ± 4.9 mm), respectively. The midpoint between the CoGs of the FCR and ECR muscles was located at (5.5 ± 7.7 mm, 52.5 ± 4.8 mm), and the coil was placed at each participant’s midpoint between the two CoGs. Vibratory stimulation evoked illusory movements in all participants. After the 6-min interval, the illusory movements were diminished in all participants.

**Figure 3:**
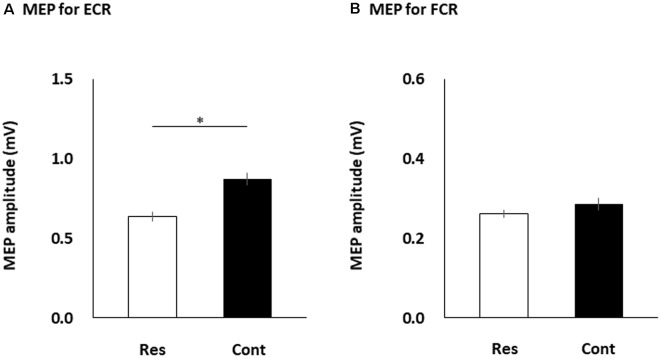
The MEP amplitudes of the ECR **(A)** and FCR **(B)** muscles during the resting and control conditions. The columns and error bars denote the means and standard errors of the mean, respectively. The ECR MEP amplitudes significantly increased in the control condition compared to the resting condition (*p < 0.0001). However, there was no significant change in the FCR MEP amplitudes between the control and resting conditions (*p* = 0.176). MEP, motor evoked potential; FCR, flexor carpi radialis; ECR, extensor carpi radialis; Res, resting condition; cont, control condition.

Table 1 shows each participant’s MEP amplitudes obtained from the ECR and ECR muscles in the four multisensory conditions (resting, control, static, and dynamic). Figure 3 shows the differences in FCR and ECR MEP amplitudes between the resting and control conditions. The paired *t*-test showed that the MEP amplitudes obtained from the ECR muscle significantly increased in the control condition compared to the resting condition (*p* < 0.0001; Figure 3A). However, there was no significant change in the MEP amplitudes obtained from the FCR muscle between the control and resting conditions (*p* = 0.176; Figure 3B).

**Table 1:** MEP amplitudes corresponding to the multisensory conditions.

Participants	MEP amplitudes (mV)							
	Rest		Control		Static		Dynamic	
	ECR	FCR	ECR	FCR	ECR	FCR	ECR	FCR
1	1.24 ± 0.10	0.23 ± 0.04	1.08 ± 0.15	0.27 ± 0.07	1.82 ± 0.11	0.07 ± 0.00	0.63 ± 0.13	0.11 ± 0.03
2	0.70 ± 0.08	0.51 ± 0.03	1.35 ± 0.12	0.42 ± 0.04	1.81 ± 0.11	0.48 ± 0.04	1.23 ± 0.10	0.56 ± 0.07
3	2.52 ± 0.11	0.39 ± 0.04	1.26 ± 0.13	0.40 ± 0.04	0.95 ± 0.07	0.55 ± 0.05	1.06 ± 0.05	0.88 ± 0.15
4	1.40 ± 0.07	0.49 ± 0.03	2.83 ± 0.14	0.27 ± 0.01	3.23 ± 0.13	0.27 ± 0.01	2.44 ± 0.13	0.27 ± 0.01
5	0.72 ± 0.08	0.27 ± 0.03	1.76 ± 0.07	0.28 ± 0.02	1.82 ± 0.12	0.30 ± 0.02	0.79 ± 0.13	0.27 ± 0.02
6	0.31 ± 0.02	0.10 ± 0.01	0.33 ± 0.05	0.08 ± 0.01	0.28 ± 0.03	0.04 ± 0.00	0.32 ± 0.03	0.09 ± 0.01
7	0.56 ± 0.05	0.10 ± 0.01	0.80 ± 0.09	0.23 ± 0.03	1.58 ± 0.14	0.27 ± 0.02	0.71 ± 0.03	0.39 ± 0.06
8	0.22 ± 0.02	0.30 ± 0.06	0.44 ± 0.05	0.12 ± 0.02	0.24 ± 0.03	0.42 ± 0.07	0.23 ± 0.02	0.50 ± 0.05
9	0.53 ± 0.04	0.40 ± 0.06	0.69 ± 0.08	0.08 ± 0.01	0.35 ± 0.04	0.09 ± 0.01	0.63 ± 0.10	0.11 ± 0.01
10	0.29 ± 0.02	0.32 ± 0.03	0.83 ± 0.08	0.24 ± 0.02	0.94 ± 0.07	0.17 ± 0.02	0.92 ± 0.09	0.22 ± 0.02
11	0.51 ± 0.03	0.10 ± 0.01	1.26 ± 0.13	0.23 ± 0.02	1.33 ± 0.10	0.19 ± 0.01	1.12 ± 0.12	0.21 ± 0.02
12	0.62 ± 0.06	0.38 ± 0.03	0.45 ± 0.05	0.30 ± 0.02	0.49 ± 0.04	0.44 ± 0.02	0.24 ± 0.03	0.22 ± 0.03
13	0.48 ± 0.05	0.17 ± 0.02	0.58 ± 0.03	0.12 ± 0.01	0.57 ± 0.04	0.13 ± 0.01	0.63 ± 0.03	0.11 ± 0.01
14	0.27 ± 0.01	0.14 ± 0.01	0.51 ± 0.04	0.09 ± 0.01	0.36 ± 0.05	0.13 ± 0.01	0.55 ± 0.09	0.13 ± 0.01
15	0.28 ± 0.03	0.14 ± 0.01	0.18 ± 0.01	0.18 ± 0.02	0.18 ± 0.03	0.22 ± 0.02	0.19 ± 0.01	0.30 ± 0.03
16	0.47 ± 0.04	0.14 ± 0.01	0.83 ± 0.06	0.23 ± 0.03	3.68 ± 0.12	0.29 ± 0.01	0.72 ± 0.03	0.27 ± 0.02
17	0.20 ± 0.02	0.12 ± 0.02	0.64 ± 0.02	1.18 ± 0.14	0.69 ± 0.02	1.40 ± 0.15	0.74 ± 0.02	1.76 ± 0.14
18	0.49 ± 0.06	0.49 ± 0.04	0.29 ± 0.06	0.40 ± 0.02	0.21 ± 0.03	0.31 ± 0.03	0.18 ± 0.01	1.32 ± 0.05
19	0.45 ± 0.04	0.13 ± 0.03	0.25 ± 0.05	0.19 ± 0.04	0.22 ± 0.05	0.13 ± 0.04	0.46 ± 0.08	0.55 ± 0.10
20	0.53 ± 0.04	0.33 ± 0.04	1.11 ± 0.04	0.42 ± 0.03	0.99 ± 0.08	0.27 ± 0.04	0.45 ± 0.03	0.18 ± 0.03
Total	0.64 ± 0.03	0.26 ± 0.01	0.87 ± 0.04	0.29 ± 0.01	1.09 ± 0.05	0.31 ± 0.02	0.71 ± 0.03	0.42 ± 0.02

*Values are mean ± standard error of the mean. MEP, motor evoked potential; ECR, extensor carpi radialis; FCR, flexor carpi radialis*.

**Figure 4:**
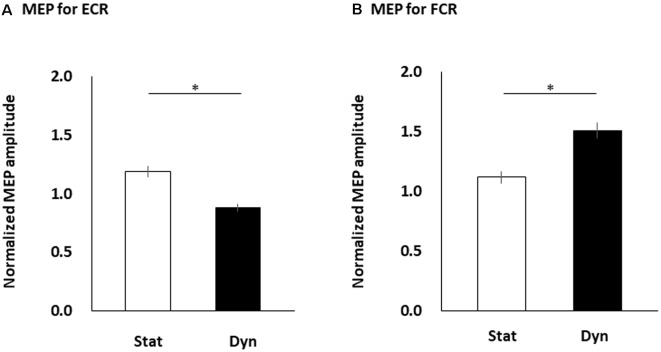
The MEP amplitudes of the ECR **(A)** and FCR **(B)** muscles during the static and dynamic conditions. The columns and error bars denote the means and standard errors of the mean, respectively. Normalized MEP amplitude was calculated by that each MEP amplitude in static and dynamic conditions divided by mean MEP amplitude in control condition in each participant’s FCR or ECR. The normalized ECR MEP amplitudes significantly increased in the static condition compared to the dynamic condition (*p < 0.0001). However, the normalized FCR MEP amplitudes significantly increased in the dynamic condition compared to the static condition (*p < 0.0001). MEP, motor evoked potential; FCR, flexor carpi radialis; ECR, extensor carpi radialis; Stat, static condition; dyn, dynamic condition.

Figure 4 shows the differences in the normalized FCR and ECR MEP amplitudes between the static and dynamic conditions. The paired *t*-test showed that the normalized MEP amplitudes obtained from the ECR muscle significantly increased in the static condition compared to the dynamic condition (*p* < 0.0001; Figure 4A). However, the normalized MEP amplitudes obtained from the FCR muscle significantly increased in the dynamic condition compared to the static condition (*p* < 0.0001; Figure 4B).

## Discussion

To test the hypothesis that multisensory difference between proprioceptive and visual information would produce unequal MEP amplitudes in reciprocal muscles, we measured changes in the corticospinal excitability of reciprocal muscles during a visuo-proprioceptive stimulation task. Our results showed that: (a) the ECR MEP amplitudes increased during the control condition rather than the resting condition, but the FCR MEP amplitudes did not; and (b) the ECR MEP amplitudes further increased during the static condition rather than the dynamic condition, but the FCR MEP amplitudes further increased during the dynamic condition rather than the static condition. Many previous reports have shown that vibratory proprioceptive stimulation increases the MEP amplitude evoked in the muscle stimulated by vibration (Rosenkranz and Rothwell, 2003; Rosenkranz et al., 2003; Lapole et al., 2015; Souron et al., 2018), and this increase is considered to be due to increased excitability of spinal mechanisms (Eklund and Hagbarth, 1966; Hagbarth et al., 1980; Claus et al., 1988). In the present study, the ECR MEP amplitudes increased during the control condition, consistent with the findings of previous studies, and probably this increment was mainly due to spinal Ia-α loop excitation by tendon vibration. However, some previous studies have reported that the MEP of the antagonist muscle decreased during muscle vibration (Rosenkranz and Rothwell, 2003; Rosenkranz et al., 2003), whereas other studies did not observe antagonist inhibition during muscle vibration (Rosenkranz et al., 2000; Mancheva et al., 2014). Previous studies that applied vibratory stimulation of 80-Hz frequency to the tendon only noted increment of corticospinal excitability for the vibratory muscle (Forner-Cordero et al., 2008). Therefore, the change in corticospinal excitability of antagonist muscles during vibratory stimulation remains controversial. In our study, the FCR MEP amplitudes did not change between the resting and control conditions. A previous anatomical study noted that Ia inhibitory interneurons had slender, weakly branching dendrites (Jankowska and Lindström, 1972). Although we cannot explain the exact reason why antagonist FCR MEP amplitudes did not decrease by vibratory proprioceptive stimulation, one possibility is that applying a vibratory stimulus to the tendon alone is insufficient to inhibit α motoneurons for antagonist muscles *via* Ia inhibitory interneurons.

In our study, all participants perceived an illusory wrist flexion movement by vibratory stimulation, although the participants’ actual hand was immobile throughout the tendon vibration. Therefore, the participants could be viewing the static or dynamic virtual hand with proprioceptive flexing sensation. This would allow for successful observation of changes in corticospinal excitabilities of reciprocal muscles by visuo-proprioceptive information difference. We observed that the ECR MEP amplitudes were further increased during the static condition (i.e., with difference between visual and proprioceptive information) rather than the dynamic condition (i.e., without difference between visual and proprioceptive information), whereas the FCR MEP amplitudes further increased during the dynamic condition rather than the static condition. In a previous functional magnetic resonance imaging (fMRI) study on proprioceptive illusory movement, the cortical activities of the SMA, M1, primary sensory cortex, and inferior parietal lobule were associated with illusory movements (Casini et al., 2006). In addition, another fMRI study on movement observation showed that the PM and inferior and superior parietal lobules were associated with observation of movements (Filimon et al., 2007; Orr et al., 2008). These imply that the neural substrates activated during illusion based on proprioceptive information and during observation based on visual information anatomically coincide. Moreover, another fMRI study (Hagura et al., 2007) showed that the human posterior parietal cortex was involved in the multisensory processing between visual and proprioceptive information and mediated visual information dominance. Animal studies have revealed that the parietal cortex is linked to the PM and SMA (Petrides and Pandya, 1984; McGuire and Sabes, 2011); thus, visual and proprioceptive inputs are conveyed from the parietal cortex to the M1 *via* the PM. One possibility is that output from the parietal cortex, PM, and SMA may influence M1 excitability with different “gains” to reduce the multisensory difference. The difference between visual and proprioceptive information occurred in the static condition because the participant who perceived the illusory wrist flexion sensation by tendon vibration was visually viewing the static virtual hand. Hagura et al. (2007) showed that the illusory movement of the vibrated hand was attenuated when the participants viewed a static hand, and the posterior parietal cortex is related to the attenuation of illusory movement. They (Hagura et al., 2007) also suggested that when both visual and proprioceptive information of a limb is available, vision is usually the dominant source of information used to perceive the spatial location. Presumably, activation of the ECR muscle could be a countermeasure for the proprioceptive illusory wrist flexion sensation to maintain the visual position of the wrist as static. Therefore, we predict that M1 excitability for the ECR muscle during the static condition could have been heightened by the difference of proprioceptive and visual information. Sensorimotor adaptation functionally involves updating an efference copy that estimates the sensory consequences of motor commands (Shadmehr and Mussa-Ivaldi, 1994; Lei et al., 2017). A previous study (Witham et al., 2010) suggested that M1 contributes to the modification of the efference copy, and M1 excitability changes induced by sensorimotor tasks generally take the form of increased excitability (Rosenkranz et al., 2007; Smyth et al., 2010). Therefore, in our study, the functional utility of the increment of M1 excitability for the ECR muscle might relate to updating the efference copy in response to a modification to the expected proprioceptive and visual signals. However, in the dynamic condition, no difference occurred between visuo-proprioceptive information because the participant who perceived the illusory wrist flexion sensation by tendon vibration was viewing the virtual flexing hand. In fact, the FCR MEP amplitudes further increased during the dynamic condition rather than the static condition. Previous studies have noted that the MEP amplitude increased during action observation (Fadiga et al., 2005; Avenanti et al., 2007; Wright et al., 2016). Because multisensory difference did not occur in the dynamic condition, the corticospinal excitability projecting to the FCR muscle might have increased by observation of the wrist flexion movement. Therefore, this increment of the FCR MEP amplitudes might be affected by observation of wrist flexion movement.

A possible limitation of this study is that our experimental paradigm does not fully distinguish between spinal and cortical effects by vibratory stimulation. In fact, vibratory stimulation may affect both spinal excitability and cortical excitability because all participants perceived illusory movements. Especially, the FCR MEP amplitudes did not increase in the control condition even though participants perceived illusory wrist flexion. Previous studies (Tsukazaki et al., 2012; Wright et al., 2014) reported that combined observation and imagery produced larger MEP amplitudes than either action observation or imagery alone. In the control condition of our study, participants did not observe wrist flexion movement (they observed a mosaic patterned static videoclip) even though participants perceived illusory wrist flexion. Therefore, spinal Ia inhibitory interneurons might be influenced by cortical descending inputs. To clarify the spinal and cortical mechanisms related to visual and proprioceptive information, future studies should consider performing a detailed examination using TMS, peripheral nerve stimulation, and brain imaging methods. In addition, previous studies have suggested that therapy using motor imagery (Tabernig et al., 2018; Geiger et al., 2019) or vibration (Ahn et al., 2019; Toscano et al., 2019) could be beneficial in the neurorehabilitation of patients with stroke. Although we investigated whether the corticospinal excitabilities for reciprocal muscles were differentially affected by visuo-proprioceptive tasks, further studies should investigate whether engaging in combined observation and vibration can facilitate motor recovery of reciprocal muscles in patients.

In conclusion, we found that visuo-proprioceptive tasks with competing multisensory information differentially altered corticospinal excitability for reciprocal muscles, and the difference between visual and proprioceptive information had a stronger effect on corticospinal excitability for the muscle to reduce the difference. These findings are potentially important for training practice with vibration stimulation and vision in rehabilitation.

## Data Availability Statement

Raw data were generated at Saitama Prefectural University. Derived data supporting the findings of this study are available from the corresponding authors, TS and MS.

## Ethics Statement

The studies involving human participants were reviewed and approved by The Ethics Committee of the Saitama Prefectural University. The patients/participants provided their written informed consent to participate in this study.

## Author Contributions

TS and MS participated in the design of the study, carried out the experiment, performed the statistical analyses, and drafted the manuscript. NK conceived of the study, participated in its design, and drafted the manuscript. TH conceived of the study, participated in its design, carried out the experiment, and drafted the manuscript. All authors read and approved the final manuscript.

## Conflict of Interest

The authors declare that the research was conducted in the absence of any commercial or financial relationships that could be construed as a potential conflict of interest.

## Abbreviations

CoG: center of gravity
ECR: extensor carpi radialis
EMG: electromyography
FCR: flexor carpi radialis
MEP: motor evoked potential
M1: primary motor cortex
PM: premotor cortex
RMT: resting motor threshold
SD: standard deviation
SMA: supplementary motor area
TMS: transcranial magnetic stimulation.
